# Toxicity of Volatile Methylated Species of Bismuth, Arsenic, Tin, and Mercury in Mammalian Cells *In Vitro*

**DOI:** 10.1155/2011/503576

**Published:** 2011-10-05

**Authors:** E. Dopp, U. von Recklinghausen, J. Hippler, R. A. Diaz-Bone, J. Richard, U. Zimmermann, A. W. Rettenmeier, A. V. Hirner

**Affiliations:** ^1^Institute of Hygiene and Occupational Medicine, University of Duisburg-Essen, Hufelandstraße 55, 45122 Essen, Germany; ^2^Institute of Environmental Analytical Chemistry, University of Duisburg-Essen, Universitaetsstraße 3-5, 45141 Essen, Germany

## Abstract

The biochemical transformation of mercury, tin, arsenic and bismuth through formation of volatile alkylated species performs a fundamental role in determining the environmental processing of these elements. While the toxicity of inorganic forms of most of these compounds are well documented (e.g., arsenic, mercury) and some of them are of relatively low toxicity (e.g., tin, bismuth), the more lipid-soluble organometals can be highly toxic. In the present study we investigated the cyto- and genotoxicity of five volatile metal(loid) compounds: trimethylbismuth, dimethylarsenic iodide, trimethylarsine, tetramethyltin, and dimethylmercury. As far as we know, this is the first study investigating the toxicity of volatile metal(loid) compounds *in vitro*. Our results showed that dimethylmercury was most toxic to all three used cell lines (CHO-9 cells, CaCo, Hep-G2) followed by dimethylarsenic iodide. Tetramethyltin was the least toxic compound; however, the toxicity was also dependend upon the cell type. Human colon cells (CaCo) were most susceptible to the toxicity of the volatile compounds compared to the other cell lines. We conclude from our study that volatile metal(loid) compounds can be toxic to mammalian cells already at very low concentrations but the toxicity depends upon the metal(loid) species and the exposed cell type.

## 1. Introduction

Biomethylation of metals and metalloids by microorganisms is a widespread phenomenon in anaerobic habitats including waste deposits, sewage sludge, and alluvial soils [1–3]. The stepwise methylation results in both partly methylated nonvolatile species as well as fully methylated volatile metal(loid) compounds. Considering the direct exposure to humans, the formation of volatile metal(loid) compounds by the intestinal biocenosis has attained considerable attention in the last years [4]. *In vivo* studies showed that after ingestion of bismuth subcitrate, the metal will be methylated by microbes in the gut and volatile trimethylbismuth (Me_3_Bi) can be detected in blood and breath [5]. Furthermore, arsenic, selenium, tellurium, and antimony were volatilized by the microbiocenosis of an *in vitro* model of the human intestinal microbiota [6]. Hollmann et al. have shown that colloidal bismuth subcitrate (CBS) as well as bismuth cysteine is methylated by human liver cells *in vitro* [7]. 

Whereas for the toxicity of nonvolatile methylated metal(loid) species, research has successively intensified in particular for arsenic [8–12] and mercury [13–15], little conclusive data are available in case of volatile species. The genotoxicity of volatile arsines has been a subject of several studies. Dimethylarsine (Me_2_AsH) induced DNA damage in human embryonic cells by formation of a peroxyl radical (CH_3_)_2_AsOO_∗_ [16]. Furthermore, Kato et al. showed that trimethylarsine (Me_3_As) induced micronuclei in the bone marrow of mice after intraperitoneal injections of 8.5 and 14.7 mg/kg [17]. These findings were confirmed by Andrewes et al. [18] who investigated the DNA-damaging potential of Me_2_AsH and Me_3_As using supercoiled DNA. They concluded that the latter two arsines are about 100 times more potent than the most genotoxic nonvolatile arsenical, dimethylarsinous acid (Me_2_AsOH). 

In comparison to nonvolatile species, volatile compounds demand a more complex experimental design and careful handling of the substances. Moreover, most studies focus on the toxicity of one compound or several compounds from one element, which makes a comparison between volatile organometal(loid) species difficult due to the different experimental systems used. 

In this study, we aimed to comparatively investigate the cytotoxic and genotoxic effects of the volatile metal(loid) compounds trimethylbismuth (Me_3_Bi), dimethylarsenic iodide (Me_2_AsI), trimethylarsine (Me_3_As), tetramethyltin (Me_4_Sn), and dimethylmercury (Me_2_Hg). 

For our studies, we developed an exposure system dedicated for the exposure to volatile organometal(loid) species. Three different cell types were chosen for toxicity testing: CHO-9 cells—an established cell system for toxicity testing, CaCo cells—human colon cells, and HepG2 cells—human hepatic cells. Same cell types were used in previous studies investigating cellular uptake and toxicity of nonvolatile organic and inorganic metal(loid) compounds [19–23]. To the best of our knowledge, this is the first study testing the toxicity of these volatile metal(loid) species *in vitro*.

## 2. Material and Methods

### 2.1. Cell Cultures

#### 2.1.1. Human Hepatoma Cells

(HepG2) (ATCC, HB 8065) were cultured in minimal essential medium (MEM) with Earle's BSS and sodium bicarbonate (CC, PRO, Germany) supplemented with 10% heat-inactivated FCS (Gibco), nonessential amino acids (0.1 mM), sodium pyruvate (1 mM), and 100 IU/mL penicillin/streptomycin (CC, PRO).

#### 2.1.2. Human Colon Cells

(CACO-2) (ATCC 169) were cultured in 75% MEM with 20% FCS, 5% nonessential amino acids (0.1 mM), 1% L-Glutamine, and 0.5% gentamycin.

#### 2.1.3. Chinese Hamster Ovary Cells

(CHO) were purchased from ECACC (UK, Cat. no. 85050302) and grown in Ham's F12 medium (CC, PRO) supplemented with 10% FCS, and 100 IU/mL penicillin/streptomycin (CC, PRO). 

All the adherent growing cell lines were kept at 37°C in a 5% CO_2_ atmosphere.

Prior to exposure approximately 2 × 10^6^ cells were placed on the membrane of cell culture inserts (ThinCerts, 0.4 *μ*m membrane, transparent; Greiner bio-one, Germany) with 3 mL of their respective medium for 24 h. 

### 2.2. Reagents (Metal(loid) Compounds)

All volatile organic metal(loid) compounds were of analytical grade unless stated otherwise and were either synthesized in the Institute of Environmental Analytical Chemistry or purchased from the following suppliers: trimethylbismuth (Me_3_Bi) from VeZerf (Idar-Oberstein, Germany), trimethylarsine (Me_3_As) from Sigma-Aldrich (Taufkirchen, Germany), tetramethyltin (Me_4_Sn) from Strem Chemicals (Kehl, Germany), and dimethylmercury (Me_2_Hg) from Acros Organics (Geel, Belgium). Dimethylarsine (Me_2_AsI) was synthesized as described in Styblo et al. [24]. Briefly, to 30 mL of an aqueous solution of dimethylarsenic acid ((CH_3_)_2_AsO(OH)) and potassium iodide (KI) concentrated sulphuric acid was added. For the reduction step, SO_2_ was bubbled through the mixture and a yellow oil ((CH_3_)_2_AsI) was separated after distillation. Identification was performed by ^1^H-NMR and GC-MS analysis (data not shown). Boiling points of all used metal(loid) species are given in Table 1.

### 2.3. Exposure of Cells

**Figure 1:**
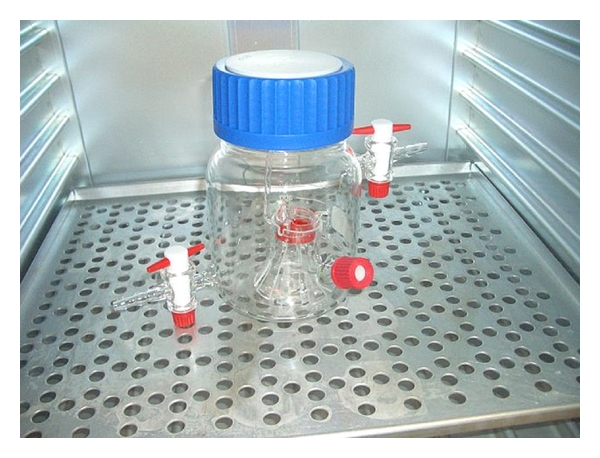
Modified glass flask for exposure of cells to volatile metal(loid) species. Cells were grown on permeable membranes. Exposure occurred through the membrane.

For exposure of cells to the volatile organometal(loid) species, the ThinCert cell culture inserts were placed in 1000 mL glass flasks equipped with a Teflon screw cap and two plug valves in order to allow purging of the gas phase. Additionally, a septum screw cap for injection of the volatile test substances was fitted at the lower end of the glass flasks. To fix a ThinCert cell culture insert into the headspace of the exposure glass flask, a suitable glass rack was designed. During exposure, the flasks were stored in an incubator at 37°C (Figure 1). The culture medium was buffered with HEPES (25 mM) (CCPro GmbH, Oberdorla, Germany).

Before exposure, the glass flask was closed and purged with argon for at least 3 minutes to purge oxygen out of the bottle because especially trimethylbismuth is extremely oxygen sensitive. Afterwards different amounts of one metal(loid) were injected through the septa screw cap and cells were exposed for 1 h. This time point was chosen because of results from previous studies which showed that longer exposure times than 1 h caused a high degree of cytotoxicity (data not shown). Exposure concentrations were chosen according to the toxicity of the volatile species. Highly toxic species required lower concentrations than non-toxic species. The concentration range was evaluated in pre-experiments (data not shown). After exposure, treated cells were harvested with trypsin (0.05%) (Sigma) for the trypan blue test and the comet assay. 

Control experiments with Me_3_As verified that the cells are exposed through the membrane and not through the culture medium, as no cytotoxic effect was observable when a nonpermeable cover was placed below the membrane (data not shown).

### 2.4. Trypan Blue Test

To detect cytotoxicity in exposed cell cultures, cell viability was evaluated with the trypan blue test immediately after exposure of cells. The cell suspension was mixed with an equivalent volume of 0.4% trypan blue solution (Sigma) and subsequently evaluated under the light microscope. The membrane of dead cells is permeable to trypan blue (blue stained cells), whereas living cells remain unstained. Cell viability is expressed as percentage of surviving cells compared to the total number of cells:

(1) $$\text{\% viable cells}=\frac{\text{unstained cells}}{\text{unstained}+\text{stained cells}}\times 100.$$

All experiments were repeated at least twice and significance was calculated by the Student's *t*-test. To compare the toxicity of the different metal(loid) compounds, LC_50_ values (lethal concentration to 50% of the cells) were calculated.

### 2.5. Alkaline Comet Assay

DNA damage was tested using the Alkaline Comet Assay, first described by Ostling and Johanson [25]. The Comet Assay is a sensitive microgel electrophoresis technique to detect DNA damage in single cells [25]. The assay was performed as described by Singh et al. [26] with minor modifications. In short, microgels were prepared by sticking a chamber slide (Chamber Slides Lab-Tek II, Nalgene Nunc International, Rochester, USA) with eight chambers to a GelBond film (Lonza GmbH, Cologne, Germany). Each chamber was sealed by adding 50 *μ*L of 0.75% low melting point (LMP) agarose (Invitrogen GmbH, Invitrogen GmbH, Germany). 45 *μ*L of LMP agarose were mixed with 20 *μ*L cell suspension containing 8,000 cells. After solidification, cells were lysed overnight at 4°C in freshly prepared lysis solution. Prior to electrophoresis, the slides were incubated in electrophoresis solution for 20 min. Electrophoresis was performed at 300 mA for 20 min and at 4°C. Then the slides were kept in neutralisation solution for 30 min and further transferred to absolute ethanol for 2 h before the gels were left to dry overnight. The DNA was stained for 15 min using SYBR Green and the extent of DNA damage was analysed at a 40x magnification using the Comet Assay IV software (Perceptive Instruments, UK) and a CCD camera attached to a Leica Microscope. Statistical analysis was done using the Mann-Whitney test. The data of three individual experiments have been summarized and are plotted using their mean value and the standard error of mean.

## 3. Results

### 3.1. Cyto- and Genotoxicity of Me_2_Hg

**Figure 2:**
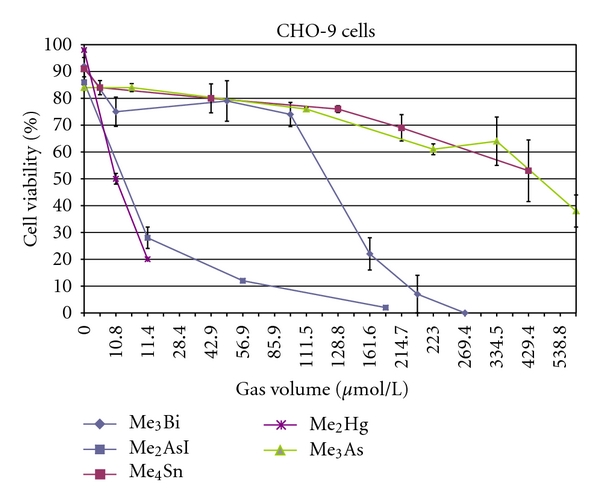
Comparison of cytotoxicity of 5 different metal(loid) compounds (Me_3_Bi, Me_4_Sn, Me_3_As, Me_2_AsI, and Me_2_Hg) in CHO-9 cells. The experiments were repeated twice.

In comparison to the tested metal(oid) compounds, Me_2_Hg was the most cytotoxic and induced 50% cell death (LC_50_) in CHO-9 cells already at the lowest concentration tested (10.8 *μ*mol/L_gv_) (Figure 2). The Comet Assay was not applicable in CHO-9 cells because the lowest tested concentration of Me_2_Hg was already cytotoxic to the cells. Due to the technical limitation of the minimal applicable droplet size, the applied concentration could not be reduced. Because of its extraordinary toxicity, not all cell lines were exposed to dimethyl mercury. Then, we abstained from exposure of the other cell lines to dimethyl mercury. The LC_50_ value for Me_2_Hg in CaCo cells was higher than in CHO-9 cells (40 *μ*mol/L_gv_), indicating a higher resistance of colon cells to the toxic compound than fibroblasts (Table 2).

### 3.2. Cyto- and Genotoxicity of Me_2_AsI and Me_3_As

**Figure 3:**
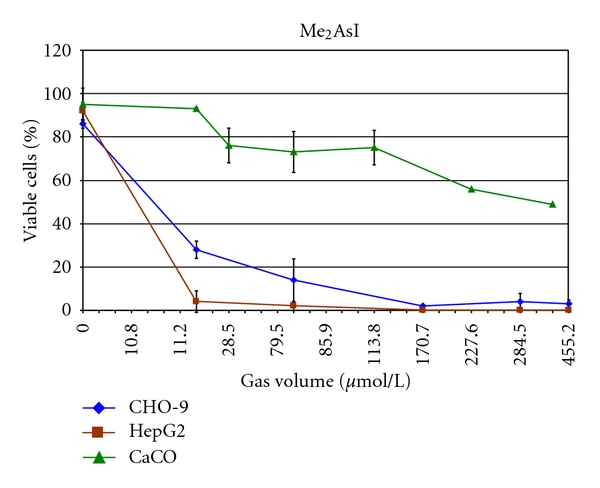
Cytotoxicity of Me_2_AsI in CHO-9, HepG2, and CaCo cells. The experiments were repeated three times.

Me_2_AsI was highly cytotoxic in HepG2 cells (LC_50_: 10.8 *μ*mol/L_gv_) and CHO-9 cells (LC_50_: 11 *μ*mol/L_gv_), whereas cytotoxicity in CaCo cells was considerably lower (LC_50_: 335 *μ*mol/L_gv_) (Figure 3, Table 2). Similar to Me_2_Hg, testing of genotoxicity was not possible because of technical limitations in application of lower concentrations.

**Figure 4:**
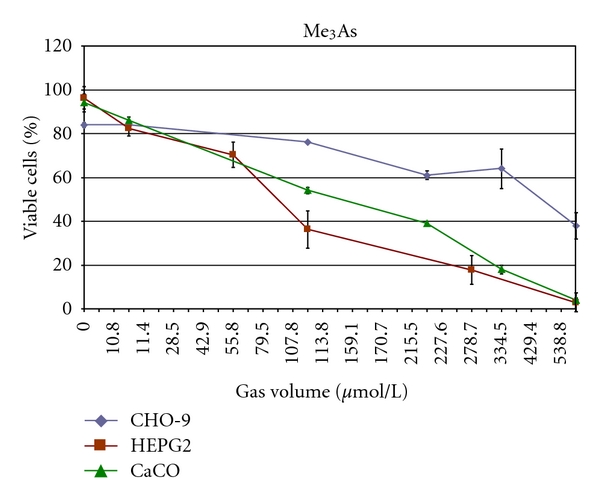
Cytotoxicity of Me_3_As in CHO-9, HepG2, and CaCo cells. The experiments were repeated three times.

**Figure 5:**
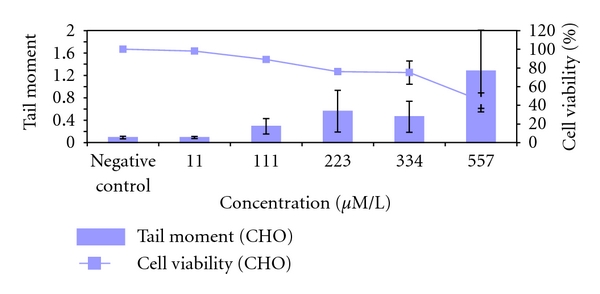
Genotoxicity of Me_3_As in CHO-9 cells after 1 h exposure time measured by Comet-Assay. The tests were repeated three times.

Me_3_As was cytotoxic in all three cell lines. HepG2 cells were most sensitive (LC_50_: 86 *μ*mol/L_gv_) followed by CaCo cells (LC_50_: 129 *μ*mol/L_gv_) and CHO-9 cells (LC_50_: 450 *μ*mol/L_gv_) (Figure 4, Table 2). There were no significant genotoxic effects in CHO-9 cells detectable up to a concentration of 334 *μ*mol/L_gv_ (Figure 5). The highest tested concentration of 557 *μ*mol/L_gv_ induced significantly elevated tail moments in the comet assay, however, the cytotoxicity was reduced below 50% in these experiments. 

### 3.3. Cyto- and Genotoxicity of Me_3_Bi

**Figure 6:**
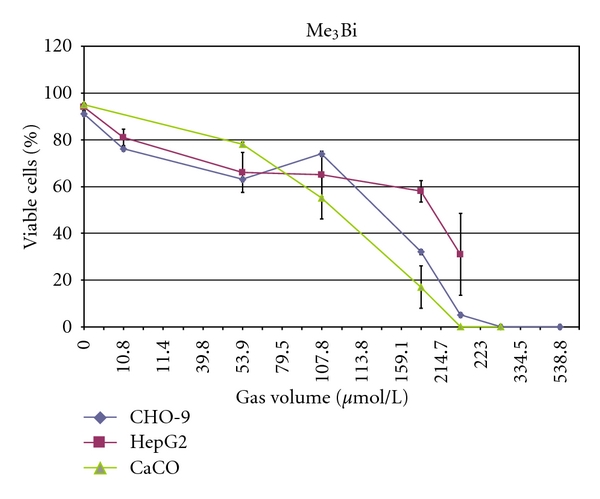
Cytotoxicity of Me_3_Bi in CHO-9, HepG2, and CaCo cells. The experiments were repeated three times.

The volatile Me_3_Bi was cytotoxic in all three tested cell lines (Figure 6).

**Figure 7:**
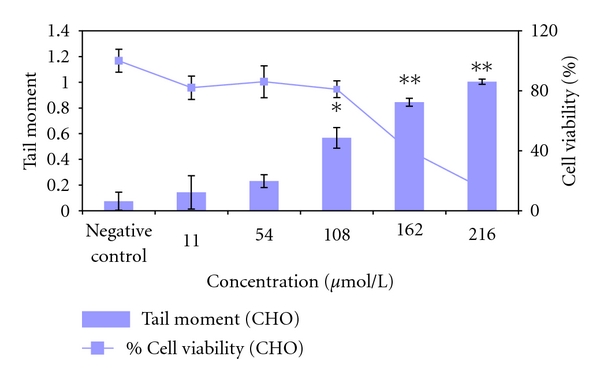
Genotoxicity of Me_3_Bi in CHO-9 cells after 1 h exposure time measured by Comet-Assay. The tests were repeated three times. *P ≤ 0.05; **P ≤ 0.01.

CaCo cells were the most sensitive cell line (LC_50_: 110 *μ*mol/L_gv_), followed by CHO-9 cells (LC_50_: 128 *μ*mol/L_gv_) and HepG2 cells (LC_50_: 194 *μ*mol/L_gv_) (Table 2). Results of the Comet-Assay revealed that Me_3_Bi was genotoxic at concentrations >108 *μ*mol/L_gv_ (Figure 7). However, at higher concentrations (162 and 216 *μ*mol/L_gv_) Me_3_Bi was cytotoxic and thus genotoxic results were not evaluable anymore.

### 3.4. Cyto- and Genotoxicity of Me_4_Sn

**Figure 8:**
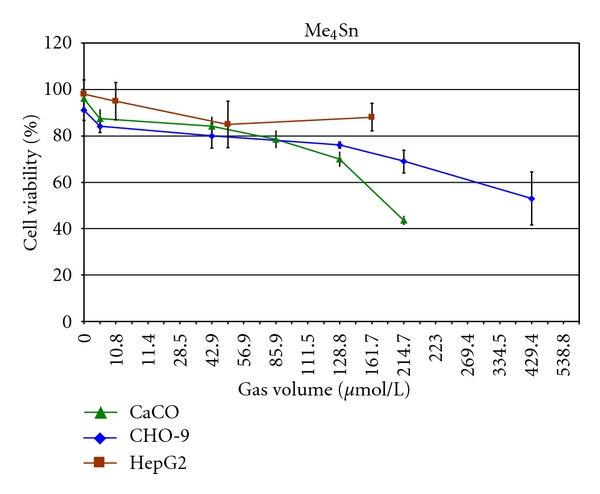
Cytotoxicity of Me_4_Sn in CHO-9, HepG2, and CaCo cells. The experiments were repeated three times.

Me_4_Sn did not show a high level of cytotoxicity and induced 50% cell death (LC_50_) just in CaCo cells at a concentration of 170.7 *μ*mol/L_gv_. In CHO-9 and HepG2 cells, the cell viability was not reduced below 50% up to a tested concentration of 429.4 *μ*mol/L_gv_ and 161.7 *μ*mol/L_gv_, respectively (Figure 8, Table 2). 

**Figure 9:**
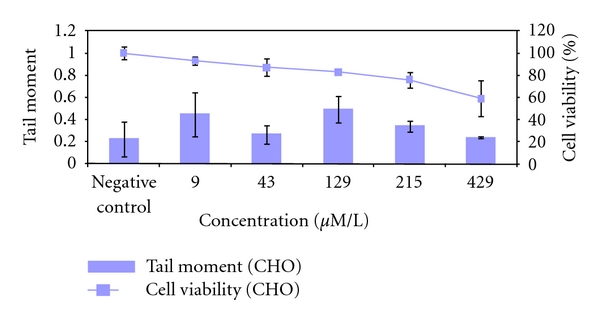
Genotoxicity of Me_4_Sn in CHO-9 cells after 1 h exposure time measured by Comet-Assay. The tests were repeated three times.

Genotoxic effects in CHO-9 cells measured by Comet-assay were not significantly elevated after Me_4_Sn exposure compared to the untreated control (Figure 9).

## 4. Discussion

From the metal(loid)s tested in this study, mercury is undoubtedly the most intensively investigated species, but this applies only to elemental and monomethyl mercury but not to the dimethylated species. In our study, Me_2_Hg was highly cytotoxic in CHO-9 and CaCo cells. Further studies regarding genotoxicity were not possible because of the high toxicity of Me_2_Hg. The extraordinary toxicity of dimethylmercury is at least known since the death of Karen Wetterhahn in 1997, months after spilling no more than a few drops of this compound on her latex-gloved hand [27]. The reason for its extraordinary toxicity is the ability of this lipophilic compound to penetrate the cell membrane. Numerous studies have implicated a molecular mimicry in the uptake of thiol conjugates in selective target cells [28]. Ehrenstein et al. reported a negligible mercury concentration of mercury inside CHO cells after treatment with dimethyl-mercury [29]. The authors suggest from their study that the volatile mercury species escapes from the treatment solution before it can pass the cell membrane. In our experimental setup, the cells are directly and continuously exposed to the gaseous compound. 

The toxicity of the volatile arsenic compounds Me_2_AsI and Me_3_As were studied in the present experiments. In both cell lines (CHO-9 and CaCo-2 cells), Me_2_AsI exhibited a very high cytotoxicity similar to Me_2_Hg. In comparison to nonvolatile Me_2_AsOH, which is among the most toxic arsenic species reported [12], similar levels of toxicity were found when comparing the LC_50_-concentrations of gas (gv) and liquid (lv) volumes, respectively (Table 3). Furthermore, Me_3_As showed a significant cytotoxicity and genotoxic effects in contrast to the nonvolatile pentavalent form, Me_3_AsO, which was not cytotoxic at the concentrations tested (Table 3).

Unexpectedly, we found significant differences between the different cell lines used. In particular, the cytotoxicity of CaCo cells towards Me_2_AsI (LC_50_: 335 *μ*mol/L_gv_) was a factor of 30 lower than that found in CHO-9 and HepG2 cells. Contrary to Me_2_AsI, CHO-9 cells were a factor of 4 to 5 less susceptible to Me_3_As than CaCo and HepG2 cells, respectively. The low susceptibility of CaCo towards Me_2_AsI could be attributed to the ability of CaCo cell to express MRP2, a multidrug resistance protein capable of catalysing as efflux [30]. The different behaviours of Me_2_AsI and Me_3_As indicate different mechanisms of their toxicological action. 

Methylated arsenic (III) species have been shown to be genotoxic in several test systems [18, 31–33] and are potent clastogens [34]. In the present experiments, we could not evaluate the genotoxicity of Me_2_AsI and Me_3_As because of its cytotoxicity at minimal applicable concentrations. Me_3_As showed significantly elevated tail moments only at cytotoxic concentrations, thus a genotoxicity testing was also not possible. 

The nonvolatile bismuth species monomethylbismuth was already tested for cyto- and genotoxicity in human cells in an earlier study [23]. The results showed that the trivalent monomethylbismuth (MeBi(III)) exerted cytotoxicity even in micromolar concentrations in human hepatocytes (LC_50_: 350 *μ*M) after 1 h exposure. In the present study, the cytotoxic effect of the volatile Me_3_Bi in CaCo, CHO, and HepG2 cells confirmed the observation that methylated bismuth compounds are more toxic than inorganic bismuth compounds. The LC_50_ value in HepG2 cells was 194 *μ*mol/L_gv_ for Me_3_Bi compared to 350 *μ*mol/L_lv_ for MeBi(III).

There seems to be a trend to an increased toxicity of methylated Bi compounds with augmented methyl groups in HepG2 cells. Cytotoxicity of a trialkylated bismuth compound has been detected until now only with triphenyl-bismuth in human embryonic lung fibroblasts [35]. In the experiments of von Recklinghausen et al. [23] with MeBi(III), the authors demonstrated that the compound is able to induce genomic damage in human lymphocytes by induction of a significant number of chromosomal aberrations and sister chromatid exchanges after 24 h exposure time. In the present experiments, we also detected DNA damage after an exposure of CHO-9 cells to Me_3_Bi for 1 hour only. Also here, Me_3_Bi seems to be more toxic than MeBi(III).

Recent studies with methylated tin compounds *in vitro* revealed a considerable toxicological potential of some organotin species but demonstrated clearly that the toxicity is modulated by the cellular uptake capability [20]. The highly hydrophobic and volatile compound Me_4_Sn induced neither cytotoxicity detected by using the trypan blue test nor genotoxicity evaluated with the comet assay in CHO-9 cells up to a tested concentration of 429 *μ*mol/L_gv_.

## 5. Summary

In summary, the present study indicates that some volatile organometal(loid) compounds are able to exhibit a significant toxicity to mammalian cells. While exposure to volatile organometal(loid)s in the environment is relatively rare, the formation of these compounds in the intestine may contribute to the toxicity of ingested metal(loid)s.

In accordance to methylated volatile arsenic species, recent studies of our group indicated that the induction of cyto- and genotoxic effects caused by the nonvolatile trivalent methylated arsenic species is primarily dependent upon their ability to penetrate the cell membrane [12]. Likewise, we assume that the high cyto- and genotoxicity for volatile organometal(loid) compounds found in this study can be attributed to their ability to pass cell membranes.

The observation that the toxicity highly depends both upon the metal(loid) species and the exposed cell type indicates different mechanisms of their toxicological action, which need to be subject of further studies.

## Figures and Tables

**Table 1:** Boiling points of volatile metal(loid) species.

Compound	Abbreviation	Boiling point	Reference
Tetramethyltin	Me_4_Sn	78°C	(Hoeppner et al., 1964)
Dimethylarsenic iodide	Me_2_AsI	155–160°C	(Lee et al., 1923)
Trimethylarsine	Me_3_As	51–53°C	(Dyke and Jones, 1930)
Trimethylbismuth	Me_3_Bi	107.1°C	(Bamford et al., 1946)
Dimethylmercury	Me_2_Hg	92°C	(Wilde, 1949)

**Table 2:** LC_50_ values of the investigated volatile metal(loid) compounds in different cell lines (exposure time: 1 h). Concentrations are given in *μmol*/*L*_*gv*_. n.ct.: not cytotoxic in the tested concentration range, n.t.: not tested.

	CaCo	CHO-9	HepG2
Me_4_Sn	170.7	n.ct.	n.ct.
Me_2_AsI	334.5	11.2	10.8
Me_3_As	128.8	450	85.9
Me_3_Bi	110.0	128.0	194.0
Me_2_Hg	40.0	10.8	n.t.

**Table 3:** Comparison of toxicity of volatile to nonvolatile species (exposure time: 1 h; n.t.: not tested).

Volatile compound	Cell type	LC_50_ *μ*mol/L_gv_	Nonvolatile compound	Cell type	LC_50_ *μ*mol/L_lv_	Literature
Me_4_Sn	CHO	n.ct. (up to 400 *μ*M)	Me_3_SnCl	CHO	n.ct. (up to 5 mM)	[20]
Me_2_AsI	CHO	11.2	Me_2_AsOH	CHO	10	[19]
Me_2_AsI	HepG2	10.8	Me_2_AsOH	HepG2	18	[22]
Me_3_As	CHO	450	Me_3_AsO	CHO	n.ct. (up to 500 *μ*M)	[19]
Me_3_As	HepG2	85.9	Me_3_AsO	HepG2	n.ct. (up to 5000 *μ*M)	[22]
Me_3_Bi	HepG2	194	MeBi(III)	HepG2	350	[23]
Me_2_Hg	CHO	10.8	MeHgCl	CHO	n.t.	

## Abbreviations

Me_3_Bi: Trimethylbismuth
Me_2_AsI: Dimethylarsenic iodide
Me_3_As: Trimethylarsine
Me_4_Sn: Tetramethyltin
Me_2_Hg: Dimethylmercury
gv: Gas volume
lv: Liquid volume
LC_50_: Lethal concentration causing death of 50% of the cells
CaCo: Human colon cells
HepG2: Human hepatoma cells
CHO: Chinese hamster ovary cells
Me: Methyl group.
